# Assessing the Acute Toxicological Effects of *Annona muricata* Leaf Ethanol Extract on Rats: Biochemical, Histopathological, and Metabolomics Analyses

**DOI:** 10.3390/toxics11080688

**Published:** 2023-08-10

**Authors:** Siti Norliyana Zubaidi, Wasim S. M. Qadi, Syahida Maarof, Norazlan Mohmad Misnan, Halimatul Saadiah Mohammad Noor, Hamizah Shahirah Hamezah, Syarul Nataqain Baharum, Nurwahyuna Rosli, Faidruz Azura Jam, Ebtesam Al-Olayan, Chuanyi Wang, Khaoula Hellal, Nawal Buzgaia, Ahmed Mediani

**Affiliations:** 1 Institute of Systems Biology (INBIOSIS), Universiti Kebangsaan Malaysia, Kuala Lumpur 43600, Selangor, Malaysia; yanacmz1216@gmail.com (S.N.Z.);; 2 Department of Food Science, Faculty of Science and Technology, Universiti Kebangsaan Malaysia, Kuala Lumpur 43600, Selangor, Malaysia; 3 Science and Food Technology Research Centre, Malaysian Agricultural Research and Development Institute, MARDI, Serdang 43400, Selangor, Malaysia; 4 Herbal Medicine Research Centre, Institute for Medical Research, National Institutes of Health, Ministry of Health Malaysia, Shah Alam 40170, Selangor, Malaysia; 5 School of Pharmacy, Management and Science University, University Drive Off Persiaran Olahraga, Section 13, Shah Alam 40100, Selangor, Malaysia; 6 Pathology Department, Faculty of Medicine, Hospital Canselor Tuanku Muhriz, Universiti Kebangsaan Malaysia, Jalan Yaacob Latif, Bandar Tun Razak, Cheras 56000, Wilayah Persekutuan Kuala Lumpur, Malaysia; 7 Faculty of Medicine, Manipal University College Malaysia (MUCM), Jalan Padang Jambu, Bukit Baru 75150, Melaka, Malaysia; 8 Department of Zoology, College of Science, King Saud University, Riyadh 12372, Saudi Arabia; 9 School of Environmental Science and Engineering, Shaanxi University of Science and Technology, Xi’an 710021, China; 10 Department of Chemistry, Muğla University, Muğla 48121, Turkey; 11 Department of Chemistry, Faculty of Science, University of Benghazi, Qar Yunis, Benghazi 5341, Libya

**Keywords:** *A. muricata*, biochemical test, histopathology, toxicity, ^1^H NMR metabolomics

## Abstract

*Annona muricata* is a common plant used in Africa and South America to manage various types of disease. However, there is insufficient toxicological information or published standard available regarding repeated dose animal toxicity data. As part of the safety assessment, we exposed Sprague Dawley rats to an acute oral toxicity of *A. muricata*. The intent of the current study was to use advanced proton nuclear magnetic resonance (^1^H NMR) in serum and urinary metabolomics evaluation techniques to provide the in vivo acute toxicological profile of *A. muricata* leaf ethanol extract in accordance with the Organization for Economic Co-operation and Development’s (OECD) 423 guidelines. A single 2000 mg/kg dose of *A. muricata* leaf ethanol extract was administered to Sprague Dawley rats over an observational period of 14 days. The toxicity evaluation (physical and behavior observation, body weight, renal function test, liver function test and ^1^H NMR analysis) showed no abnormal toxicity. Histopathological analysis manifested mild changes, i.e., the treated kidney manifested mild hypercellularity of mesangial cells and mild red blood cell congestion. In addition, there was mild hemorrhage into tissue with scattered inflammatory cells and mild dilated central vein with fibrosis in the liver. However, the changes were very mild and not significant which correlate with other analyses conducted in this study (biochemical test and ^1^H NMR metabolomic analysis). On the other hand, urinary ^1^H NMR analysis collected on day 15 revealed high similarity on the metabolite variations for both untreated and treated groups. Importantly, the outcomes suggest that *A. muricata* leaf ethanol extract can be safely consumed at a dose of 2000 mg/kg and the LD_50_ must be more than 2000 mg/kg.

## 1. Introduction

Since the dawn of civilization, people have been applying herbal plants as medicines in the form of poultices, tinctures, elixirs, powders, and other herbal formulations to alleviate chronic illnesses or to improve their general health. Currently, this practice is becoming more common in our culture [1]. However, a lack of scientifically supported information can lead to improper use, for example, the restricted knowledge on the validity of the plant material, effectiveness, bioavailability, prescription methods, and toxicant compositions as well as the proper concentrations and mechanisms of medicinal plants may lead to fatal consequences [2]. Therefore, effective toxicity testing is crucial to assess the potential hazardous effect of any compounds before they can be considered safe for human consumption.

In recent years, extracts and active components of *Annona muricata* have gained tremendous fame among researchers due to their rich nutritional profile and extensive pharmacological activities. *A. muricata* is also known as graviola, sirsak, ‘durian belanda’, soursop and guanabana. It is from the apple-custard genus that belongs to the *Annonaceae* family [3]. Various parts of *A. muricata* have long been used to treat cancer, inflammation, diabetes, infections, hypertension, and wound injuries traditionally [4]. Leaves of *A. muricata* appear to be one of the common parts that have been made into juice and decoction for medicinal treatments such as cancer, diabetes, insomnia, cystitis, abscess reduction, asthma, flu, and analgesics [3,5]. Based on the *A. muricata* review, it is reported that the leaves show potential for anti-diabetic, anti-tumor, anti-microbial, and cytotoxic activities [3]. Alkaloids, phenols, and acetogenins are the bioactive substances found in this plant, which are possibly responsible for all these activities [6].

One of the studies testified that high intake of soursop leaves can cause extremely low blood glucose level [7]. One of the sources of toxic compounds that produce the toxicity effect is annonacin, which belongs to acetogenins group [8]. A comprehensive understanding of its toxicological effects remains limited. Specifically, little is known about the variation in endogenous metabolites after the administration of *A. muricata* leaf extract. To date, several acute toxicity profiles of *A. muricata* leaf extract have been published with a variety of solvents. Heretofore, all the previous studies have outlined that it is safe to consume. However, there is no study on the endogenous metabolites variation after being administered with *A. muricata* leaf extract which holds the key towards a comprehensive understanding of its toxicity effects.

Acute, subacute, and chronic testing are used to determine a substance’s toxicity. Acute toxicity analysis is a short-term toxicity assessment employing a single dose of the test substance [9]. This testing can yield a wealth of knowledge, including safety, identification of the toxicity on the targeted organ, and monitoring guidelines for those involved in the development and testing of test substances. The guidelines may cover information on adverse effects on the environment, animals, and health, appropriate dose selection, recommendations for other testing programs, labeling, and transportation of chemical agents [9].

To the best of our knowledge this is the first study to employ a metabolomics approach in evaluating the toxicity effect of *A. muricata* extract using a high-throughput omics technique to systematically profile, quantify and integrate a large number of low-molecular-weight endogenous metabolites that reflect the pathological status of the subjected organism [10]. Rat models were used in this study in determining the acute toxicity effect of single-dose administration of 2000 mg/kg *A. muricata* leaf ethanol extract. When a high dose of extract was administered, acute toxicity tests were carried out within the first 24 h to investigate any negative effects that may manifest in the Sprague Dawley rat model [11]. Physical observation, biochemical, and histopathological analysis were carried out to achieve this purpose. Furthermore, metabolome profiles from urine and serum samples were analysed to study the possible metabolite changes after being treated with *A. muricata* leaf ethanol extracts.

## 2. Materials and Methods

### 2.1. Drugs and Chemicals

In this study, 5% Tween 20 (Systerm, Selangor, Malaysia) was used as an emulsifier and Formalin (Systerm, Selangor, Malaysia) for preserving tissue samples. The extraction process utilized 95% ethanol (Systerm, Selangor, Malaysia) diluted into 80%. Potassium dihydrogen phosphate (KH_2_PO_4_) (Merck, Darmstadt, Germany), deuterium oxide (D_2_O) (Cambridge, MA, USA), and trimethylsilylpropanoic acid (TSP) (Acros Organic, Geel, Belgium) were solutions and reagents used in preparing the samples for proton nuclear magnetic resonance (^1^H NMR) (JEOL, Tokyo, Japan) analysis. D_2_O is a solvent used in ^1^H NMR sample to disperse the molecules, so they do not interact with one another. This is because the protons of -OH and -NH molecules can easily exchange with water or acid. D_2_O is stable since its nucleus contains one neutron and one proton (^2^H or D). D_2_O molecules will rapidly exchange with -OH and -NH molecules and make the compound stable and stabilize the magnetic field strength [12]. KH_2_PO_4_ acts as a buffer to maintain the pH at a constant value of 7.4 [12]. TSP acts as a standard internal reference that worked to improve the quality of the data by calibrating the solutions using the ratio between analytes and TSP [13].

### 2.2. Plant Extract Preparation

A total of 5 kg of *A. muricata* leaves were purchased from the market. The plant was identified by Dr. Shamsul Khamis, an in-house botanist of the School of Environmental and Natural Resource Sciences, Universiti Kebangsaan Malaysia, where a voucher specimen (SK2501/19) is deposited. The sample was ground and sieved to homogenize it into particle size using an industrial blender. A volume of 95% absolute ethanol was diluted to 80% ethanol and mixed with the ground sample of *A. muricata* leaves in a proportion of 5% *w*/*v*; 50 g of samples was added into 1 L of 80% ethanol. The solution was then sonicated in ultrasonic bath sonicator for one hour at room temperature [14]. After that, the sample was filtered by using Whatman filter paper to sieve out the debris. The solution was evaporated by using rotary evaporator (Buchi, Switzerland) to vaporize the solvent and concentrate the crude extract until the solution become thick and viscous. The remaining solution was then freeze dried to eliminate the water content. The crude extracts were stored at −80 °C until used for further analysis.

### 2.3. Phytochemical Analysis

The leaf extract of *A. muricata* was subjected to phytochemical screening for the qualitative detection of flavonoids, alkaloids, saponin, tannins, steroids, triterpenes, and carbohydrate using the conventional method [15,16].

#### 2.3.1. Flavonoids Test

A small amount of *A. muricata* leaf ethanol extract was mixed with one to five drops of concentrated hydrochloric acid (HCl). The presence of flavonoids is indicated by the rapid development of a red color.

#### 2.3.2. Alkaloids Test

A 0.2 mL volume of diluted HCl was added into 2 mL of *A. muricata* leaf ethanol extract followed by 1 mL of Meyer’s reagent. Alkaloid content is indicated by a yellowish color.

#### 2.3.3. Saponins Test

The extract with an amount of 1 mL was diluted to a volume of 20 mL by using distilled water, and the mixture was mixed in a graduated cylinder for 15 min. The presence of saponins was detected when a centimeter-thick layer of foam was formed.

#### 2.3.4. Tannins Test

Ferum (II) chloride (FeCl_3_) with the concentration of 5% was added to 5 mL of *A. muricata* leaf ethanol extract. The presence of alkaloids was detected with the formation of the greenish-black precipitate.

#### 2.3.5. Steroids Test

Acetic acid at a volume of 2 mL was added into 0.2 g of *A. muricata* ethanol extract. Sulfuric acid was added to the solution after cooling it well in ice. Color development from violet to blue or bluish-green showed a positive result.

#### 2.3.6. Carbohydrate Test

The extract was dissolved with distilled water first then a few drops of Molisch’s reagent were added to the extract. Sulfuric acid (1 mL) was added to the solution by the side of the test tube. The formation of red or dull violet color at the interphase of the two layers indicates the presence of carbohydrates.

### 2.4. Animal Experimental Analysis

Adult and healthy female rats were obtained from A Sapphire Enterprise, Malaysia. This study used female rats because they are more chemically sensitive than male rats which usually easily can be portrayed when acute toxicity happens [17]. In total, 12 Sprague Dawley strain rats were used in this study with a range of weight between 190 and 260 g. All of them were at the age of 12 weeks old. Each rat was housed in a plastic cage with free access to standard rat pellets and distilled water ad libitum for four weeks for the research preparation and acclimatization purpose before the study was started. They were also maintained under constant humidity and in an air-conditioned (24 °C) environment. The animals used for the experiment were approved by the Universiti Kebangsaan Malaysia animal ethics committee (UKMAEC) (IBS/2022/AHMED MEDIANI/26-JAN./1221- JAN.-2022-AUG. -2023).

### 2.5. Acute Oral Toxicity Study

**Figure 1:**
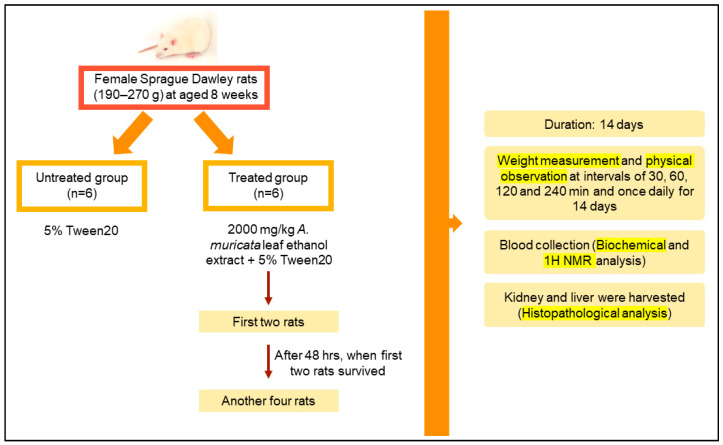
A summarized flow of acute toxicity test design.

A total of 12 rats were used over the 14-day study period and were randomly divided into two groups, treated and untreated groups containing six rats each. The use of twelve female rats for the study and the rats’ groupings were in accordance with a previous study [18]. The treated group was given a single administration of 2000 mg/kg *A. muricata* leaf ethanol extract for the acute toxicity study design. The dose was chosen based on a limit test from Organization for Economic Co-operation and Development OECD guideline 423 to determine the range of lethality dosage [19]. The *A. muricata* leaf extract was dissolved in 5% Tween 20 hence, the untreated group was given the same chemical as a control which indicates that the toxicity was not caused by the Tween 20 solution. The extract was administered once via oral gavage and the rats were fasted four hours after the dosing. The first two female rats were given a single dose of 2000 mg/kg body weight of the extracts and another four rats received the same dose of the extracts at intervals of 48 h since the first batch of rats were alive (Figure 1). After the extract administration, cage side and physical observations were carried out to assess any signs of toxicity at the interval of 30, 60, 120, and 240 min and once daily for the next days for 14 days [18]. Throughout the study, the rats were observed in order to note any abnormal symptoms including inactivity, alertness, abnormal breathing, eyes and nose discharge, diarrhea, abnormal movement, and mortality. This observation was assessed based on monitoring scoresheets taken form the Code of Practice for the Housing and Care of Laboratory Mice, Rats, guinea Pigs and Rabbits. The scoresheets were adjusted according to the necessity of the study. Their body weight was also recorded every two days. At the end of the study, all rats were euthanized with exposure to carbon dioxide gas. Urine, blood, liver and kidney were collected for further analysis.

### 2.6. Biochemical Analysis

On day 15, the rats were placed into a tight container and carbon dioxide gas was released to euthanize them. Then, the blood was collected from animals through posterior vena cava after the rats were fasted overnight. A 10 mL volume of blood was withdrawn and stored in ethylenediamine tetraacetic acid (EDTA) tube and serum separator tube (SST) with each tube containing 5 mL of blood for hematological and metabolomics analyses. Blood from SST tube was processed, and serum was collected and placed in microcentrifuge tube for metabolomics and biochemical analyses and lastly stored in −80 °C until ready for used. The collected serum was subjected for biochemical analysis by using automated chemistry analyzer (DIRUI CS-300B) at Science & Food Technology Research Centre (FT), Malaysian Agricultural Research and Development Institute, Malaysia (MARDI). The parameters included serum creatinine and blood urea nitrogen (BUN) for renal function test and alanine aminotransferase (ALT), aspartate aminotransferase (AST) and alkaline phosphatase (ALP) for liver function test.

### 2.7. Histopathological Analysis

The liver and kidney were harvested from the animals after they were sacrificed, and these organs were cleaned using saline solution. These organs were preserved in 10% buffered formalin. The organs were then processed, sectioned into 4 microns in thickness using a microtome (Leica, Wetzlar, Germany), and stained with haematoxylin and eosin. A detailed description of the staining process was that the slides were submerged in Xylene, 100% alcohol, and 70% alcohol separately with a duration of 5 min for each solution. The slides were rinsed first before submerging them in haematoxylin for 5 min. After that, the slides were rinsed 3 to 5 times. The slides were then dipped in 1% acid alcohol for 3 s and run under tap water for 5 min. Next, the slides were submerged in eosin for 1 min, sprayed with 95% alcohol, and slides were rinsed again under running tap water for 5 to 10 s. The slides were sprayed with 95% alcohol before cleaning them and left to dry. Then, the slides were mounted with dibutylphthalate polystyrene xylene (DPX) and ready for viewing. The slides were observed under a light microscope. The degree of abnormalities observed in the slides was categorized as mild, moderate, and severe.

### 2.8. Serum and Urine Sample Preparation and ^1^H NMR Acquisition and Processing

Serum samples were thawed first before centrifuging them at 3000× *g* for 10 min to remove any precipitate. A 200 µL volume of supernatant was transferred into microcentrifuge tubes and mixed with 400 µL phosphate buffer solution (0.308 g of KH_2_PO_4_, in 25 mL of D_2_O, pH 7.4 containing 0.2% TSP). The samples were vortexed to obtain a homogenous mixture. Approximately 550 µL of the mixture was then transferred into NMR tubes and ready to be used for ^1^H NMR analysis.

On the other hand, the collected urine samples were thawed and centrifuged at 3000× *g* for 10 min in order to remove any precipitate. A total of 400 µL of the supernatant was mixed with 200 µL phosphate buffer solution in a microcentrifuge tube (0.308 g of KH_2_PO_4_, in 25 mL of D_2_O (pH 7.4), containing 0.1% TSP). The samples were vortexed to obtain a homogenous mixture and then transferred into NMR tubes for ^1^H NMR analysis.

The ^1^H NMR analysis was performed using a JNM-ECZ-600R 600MHz spectrometer at Institute for Medical Research (IMR), National Institutes of Health (NIH), Malaysia. The urine sample underwent PRESAT experiment meanwhile water suppression was done by using Carr–Purcell–Meiboom–Gill (CPMG) pulse sequences to suppress large residual water resonance and broad protein resonance, which enabled the portrayal of signals from small molecules for the serum sample. The parameters utilized for this analysis were spectral width of 15 ppm, 3 s of acquisition time, 7.0 s of relaxation delay time, 90° pulse width, temperature of 293 K, and 64 scans. A single pulse sequence was used for one-dimensional ^1^H NMR experiments. The following parameters were used for this analysis: spectral width of 20 ppm; time domain data points: 16K; flip angle: 45°; relaxation delay: 5 s; spectrum size: 16K; number of scans: 32. After that, JEOL Delta processing software (Version 5.1.13) was manually applied for spectra phase. The baseline was corrected and referenced to TSP (δ 0.00).

The obtained ^1^H NMR spectra were further subjected to manual phasing, baseline correction, and a binning process by using Chenomx software (Version 8.2). The water region in which at signal of δ 4.56–5.1 was excluded. Spectral data in the region of δ 0.50 to δ 10.00 were normalized via the total spectral area normalization method. They were then binned into a bin spectral width of 0.04 ppm, generating a total of 237 integrated regions per NMR spectrum for both urine and serum samples. The data were further processed through SIMCA-P+ (Version 14.1). Pareto scaling was applied to all data.

### 2.9. Statistical Analysis

The data, including serum clinical chemistry and hematological parameters, are expressed as the mean ± SD. Statistical comparisons were performed using analysis of Student’s *t*-test. The data were analyzed using GraphPad Prism (Version 8.0.1) and SIMCA-P+ (Version 14.1) software. The criterion for statistical significance was set at *p* < 0.05.

## 3. Results

### 3.1. Phytochemical Analysis

The phytochemical analysis of the *A. muricata* extract revealed the presence of flavonoids, alkaloids, triterpenes, tannins, and steroids. However, saponins were noticeably absent (Table 1).

### 3.2. Acute Toxicity Test-Related Physical and Behavioral Changes

**Figure 2:**
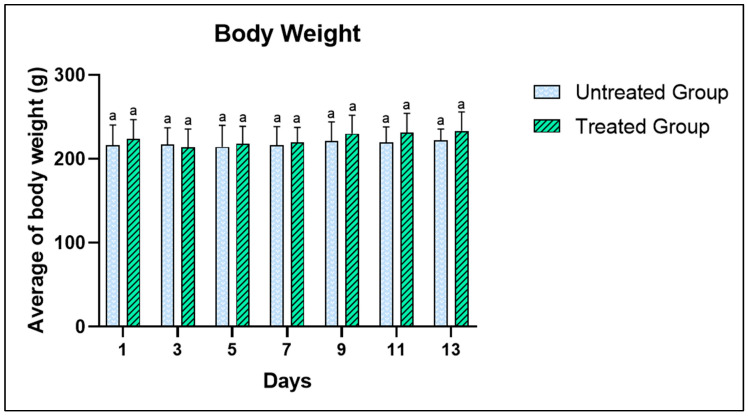
Body weight of the rats during the period of acute toxicity study when administered with 2000 mg/kg of *A. muricata* leaf ethanol extract. The values are the mean ± SD by using paired *t*-test; with n = 6 for each group. Same superscript letter (a) means no significant difference from each other (*p* > 0.05).

The acute toxicity of *A. muricata* leaf ethanol extract was evaluated to determine the toxicity of *A. muricata* leaf ethanol extract at a dose of 2000 mg/kg. Observations of the physical state and behavior were conducted after administering a single dose of 2000 mg/kg body weight of the extract. In the untreated group (5% Tween 20), no physical changes were detected. However, within the treated group, mild symptoms such as inactivity and slight incoordination were noted within 2 h post-administration, which subsided after 4 h. This could be attributed to the rats’ initial reaction to the oral gavage treatment. Future research is recommended to explore the rats’ behavioral responses to stress. No abnormal behaviors were observed in either group 4 h after being gavaged with *A. muricata* leaf ethanol extract, and this continued until the end of the study. The body weight of the rats, recorded every two days, did not show significant differences between the groups (Figure 2). The rats’ weight increased from day 3 onwards, from 223 to 233 g by the end of the 14-day study period. Importantly, no mortality was observed throughout the study.

### 3.3. Biochemical Analysis

The effects of acute toxicity of *A. muricata* on biochemical parameters are presented in Table 2 and Table 3 that evaluate on liver function test and renal function test. The parameters for liver function include aspartate aminotransferase (AST), alkaline phosphatase (ALP) and alanine aminotransferase (ALT). These parameters were compared with the untreated group by Student’s *t*-test. From the result, there were no statistically significant differences in the liver enzyme markers between treated and untreated groups. On the other hand, renal function test includes urea and creatinine enzyme level measurement. The result (Table 3) exhibited that there were no apparent alterations in these enzymes after being treated with a single dose of 2000 mg/kg of *A. muricata* leaf ethanol extract.

### 3.4. Histopathological Analysis

#### 3.4.1. Effects of The Extract on Kidney

**Figure 3:**
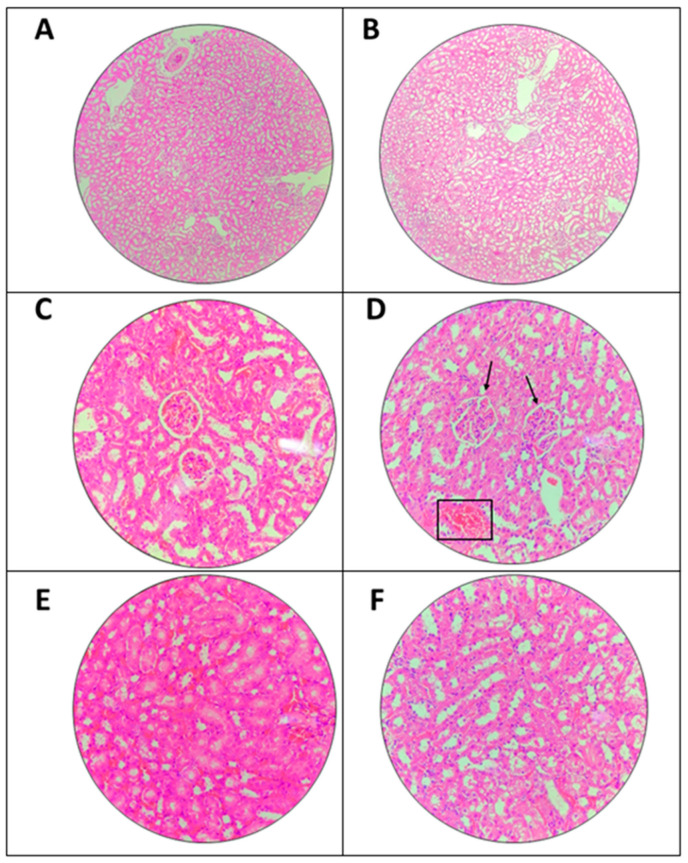
Microscopic examination of kidney in acute oral toxicity test. (**A**,**C**,**E**) Micrographs obtained from the untreated group while (**B**,**D**,**F**) from the treated group, 2000 mg/kg ethanol *A. muricata* leaf extract. (**A**,**B**) Shown at 10× magnification while (**C**–**F**) are at 40× magnification. (**D**) Mild hypercellularity of mesangial cell (↓) and mild capillary congestion (□). Other abject are showing normal histology of kidney.

Microscopic examination of kidney is shown in Figure 3. The treated group was compared with untreated group in which there were slight histological changes in single dose 2000 mg/kg of *A. muricata* leaf ethanol extract group. However, the changes were very mild and did not cause a significant toxicity effect. Vacuolation between the renal tubules in both treated and untreated group could be observed which may not happen due to extract.

#### 3.4.2. Effects of the Extract on Liver

**Figure 4:**
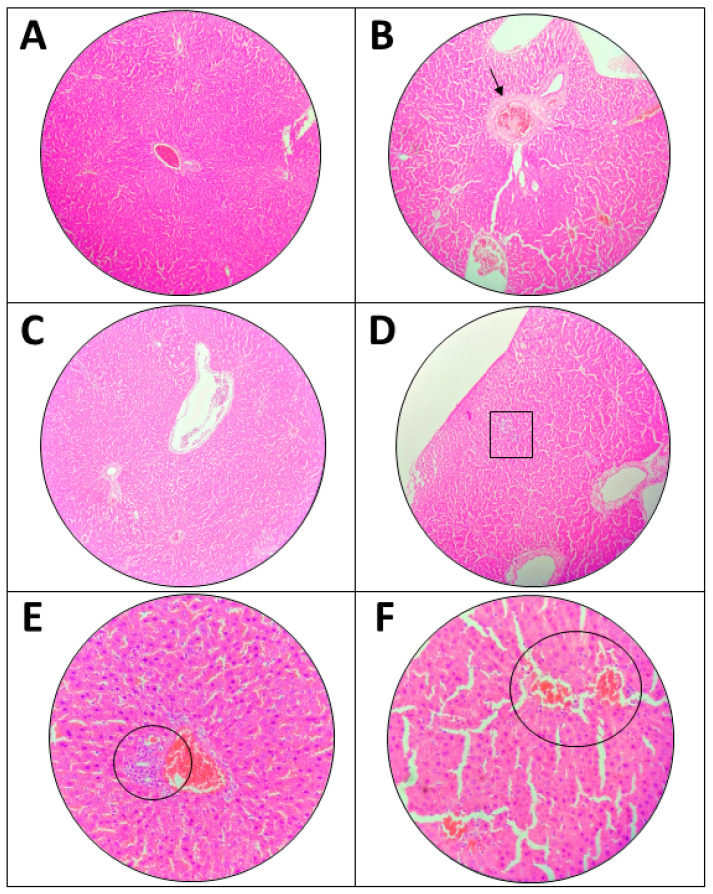
Microscopic examination of liver in acute oral toxicity test. (**A**,**C**,**E**) are from untreated group meanwhile (**B**,**D**,**F**) from treated group, 2000 mg/kg ethanol *A. muricata* leaf extract. (**A**–**D**) Shown at 10× magnification, and (**E**,**F**) at 40× magnification. (**B**) Mild dilated central vein with fibrosis (↓) and (**D**) mild infiltration of inflammatory cells (□). (**E**) Mild inflammatory cells without hemorrhage (o). (**F**) Mild hemorrhage into the tissue with scattered inflammatory cells (○).

Microscopic observation of the liver is shown in Figure 4. The comparison between untreated and treated groups (2000 mg/kg ethanol *A. muricata* leaf extract) is arranged side by side. There are several histopathological changes that were observed in the treated group, in which there was mild hemorrhage into tissue with scattered inflammatory cells, and slight infiltration of inflammatory cells in the tissue. Inflammatory cells also could be found in the untreated group. Lastly, it could be noticed in the treated group, that there was a mild dilated central vein with fibrosis whereas the central vein became slightly bigger and filled with pink dot. Moreover, the hepatocytes appeared normal, and no necrosis was found. The slight changes that occurred were very mild and the defect was not significant.

#### 3.4.3. H NMR Serum Metabolomics Evaluation

**Figure 5:**
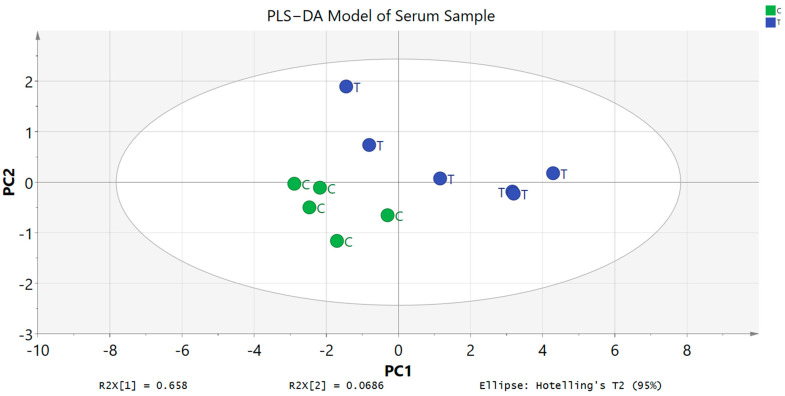
PLS−DA Model of serum profiles of the treated and untreated groups collected on day 15. C: untreated group, T: treated group.

**Figure 6:**
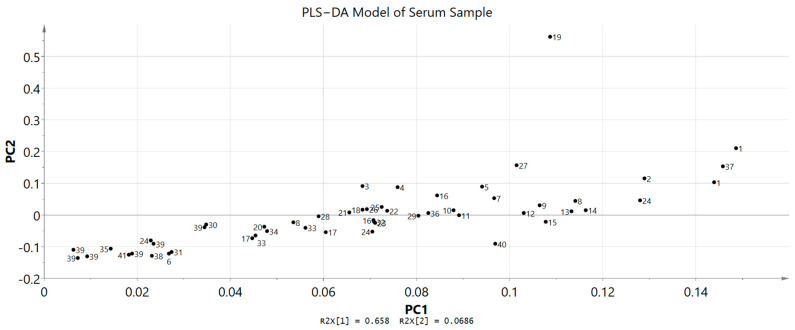
Loading Scatter Plot Model of serum profiles of the treated and untreated group collected on day 15. The scattered dots were compounds found in both groups (treated and untreated groups). 1: glucose, 2: trimethylamine *N*−oxide (TMAO), 3: arabinose, 4: ribose, 5: *O*−acetylcarnitine, 6: 2−hydroxyvalerate, 7: isoleucine, 8: *N,N*−dimethylglycine, 9: 3,4−dihydroxybenzeneacetate, 10: homovanillate, 11: arginine, 12: 5−methoxysalicylate, 13: glycine, 14: 3−methylxanthine, 15: anserine, 16: 2−hydroxy−3−methylvalerate, 17: 2−Hydroxyisovalerate, 18: nicotinamide adenine dinucleotide phosphate hydrogen (NADPH), 19: riboflavin, 20: threonate, 21: phenylalanine, 22: creatine, 23: *O*−phosphocholine, 24: acetoacetate, 25: methanol, 26: dimethyl sulfone, 27: creatinine, 28: citrate, 29: pyruvate, 30: 3−hydroxyisobutyrate, 31: 5−hydroxylysine, 32: butyrate, 33: acetoin, 34: caprate, 35: methionine, 36: *N*−acetylglucosamine, 37: acetate, 38: thymine, 39: glycocholate, 40: 2−hydroxyisobutyrate, 41: arginine.

**Figure 7:**
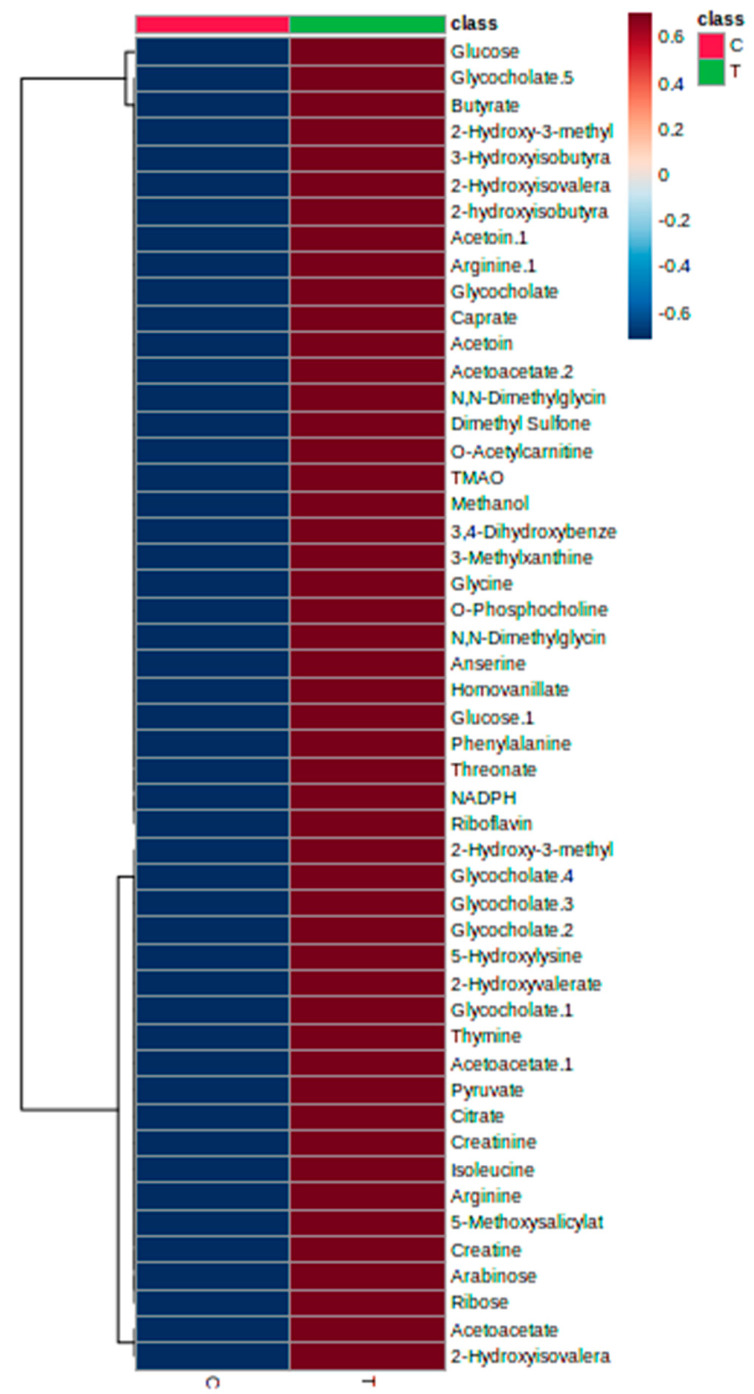
Heatmap of differential metabolites found by metabolomics analysis. The blue color represents a decreasing trend, red represents a rising trend. It represents the results of metabolic pathway in different treatment groups. C: untreated group, T: treated group.

A study regarding metabolic variations associated with the toxicity signals was carried out through ^1^H NMR spectroscopy. Serum samples from both groups, untreated and treated rats that were collected at the end of the investigation (day 15) were analysed to identify and differentiate the metabolic phenotypes and possible metabolite biomarker changes. Chenomx software databases (Version 8.2) were used along with literature data in identifying the metabolites and in total, 15 primary metabolites were successfully detected. ^1^H NMR spectra data of the untreated and treated groups did not show any significant differences via visual comparison (Figure 5 and Figure 6). Then, in order to effectively establish a pertinent summary from the ^1^H NMR spectra data and to minimize any potential biases, a multivariate data analysis (MVA) tool was combined with the data. Principal component analysis (PCA) was used on the data to examine any differences between the two groups. The score scatter plot showed that there was no clear separation in between untreated and treated groups as the compounds tend to be on the other side of the opposite group. For example, the treated group can be found on both right and left sides of PC1. Partial least squares discriminant analysis (PLS−DA) was then carried out for a better separation and the score scatter plot showed slight separation but there were still no significant differences between these two groups. Variable importance in the projection (VIP) was also used to identify the elevated metabolites after being treated with *A. muricata* leaf ethanol extract at 2000 mg/kg. The model was validated with response permutation testing and cross validation (R^2^Ycum = 0.871, Q^2^Ycum = 0.372). Both PCA and PLS−DA models showed non-significant results when compared between untreated and untreated groups. The 15 compounds that appeared slightly elevated in the treated group were glucose, trimethylamine *N*−oxide (TMAO), arabinose, ribose, *O*−acetylcarnitine, 2−hydroxyvalerate, isoleucine, *N*,*N*−dimethylglycine, 3,4−dihydroxybenzene, homovanillate, arginine, 5−methoxysalicylate, glycine, 3−methylxanthine and anserine. Even so, this elevation of compounds was not significant when compared to the untreated group (Figure 7).

### 3.5. ^1^H NMR Urinary Metabolomics Evaluation

**Figure 8:**
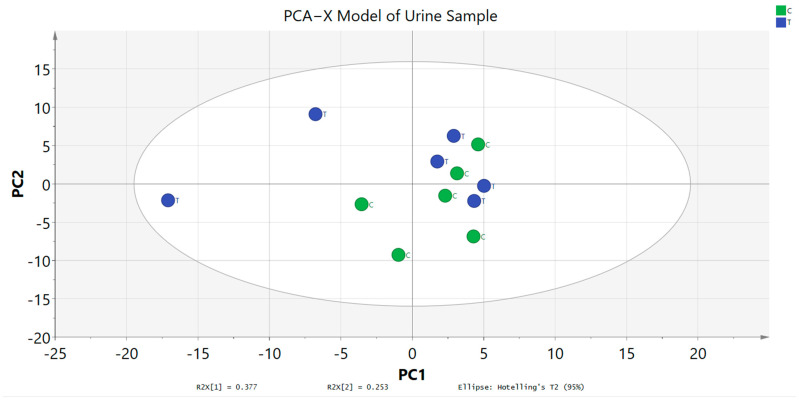
PCA−X Model of urine profiles of the treated and untreated groups collected on day 15. C: untreated group, T: treated group.

^1^H NMR spectroscopy was executed for urine samples of untreated and treated groups that were collected on day 15. The metabolites were successfully identified by using the Chenomx software databases and literature data. A general visual evaluation of the spectra suggests that there were no metabolic variations between untreated and treated groups. After that, MVA tool was used for further examination of the metabolic variations between the two groups. PCA score plot data showed that the metabolites between the two groups were clustered together and there was no separation between them (Figure 8). This result did not reveal any significant metabolic products. It also indicates that there were no significant toxic compounds produced after being administered with 2000 mg/kg of *A. muricata* leaf ethanol extract. Moreover, the metabolites variation appeared in both groups with high similarity.

**Figure 9:**
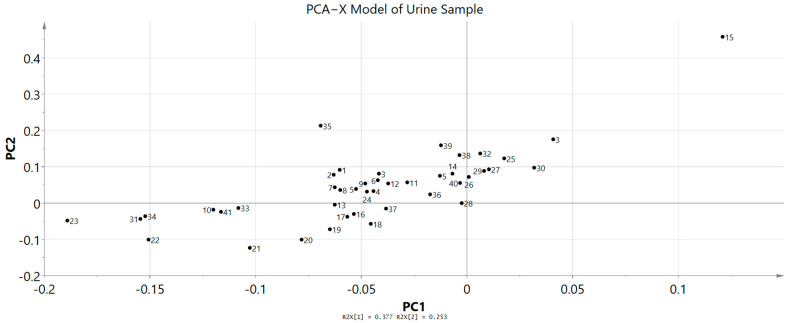
Loading Scatter Plot Model of urine profiles of the treated and untreated groups collected on day 15. The scattered dots were compounds found in both groups (treated and untreated groups). 1: Isocitrate, 2: succinate, 3: allantoin, 4: indole−3−acetate, 5: *N*−nitrosodimethylamine, 6: *N*−phenylacetylglycine, 7: pyridoxine, 8: xanthosine, 9: phenylalanine, 10: methanol, 11: fucose, 12: *N*−acetylglutamate, 13: 3−hydroxyphenylacetate, 14: cytidine, 15: urea, 16: UDP−galactose, 17: UDP−glucose, 18: UDP−*N*−acetylglucosamine, 19: glucose-1-phosphate, 20: arabinose, 21: ribose, 22: trehalose, 23: riboflavin, 24: 1−methylnicotinamide, 25: glucarate, 26: betaine, 27: arginine, 28: kynurenine, 29: indole−3−acetate, 30: acetoacetate, 31: 1,3−dimethylurate, 32: trimethylamine *N*−oxide, 33: *O*−phosphoethanolamine, 34: beta−alanine, 35: creatine, 36: methylguanidine, 37: sarcosine, 38: dimethylamine, 39: citrate, 40: 3−hydroxyisovalerate, 41: *N*−acetylcysteine.

PLS−DA loading plot model was carried out and the result remains the same in which there were no significant differences between untreated and treated groups (Figure 9). This model was executed to discriminate the patterns and detect any metabolite changes when compared between the two groups. The metabolites of untreated and treated groups closely clustered together suggest that both groups had high similarity on the metabolite variations. The model was validated with response permutation testing and cross validation. Furthermore, 12 compounds appeared slightly higher even though it was not significant which included isocitrate, succinate, allantoin, indole−3−acetate, *N*−nitrosodimethylamine, *N*−phenylacetylglycine, pyridoxine, xanthosine, phenylalanine, methanol, fucose and *N*−acetylglutamate.

## 4. Discussion

The toxicological study in this research on *A. muricata* leaves focused on the physical and behavioral changes of the rats, biochemical analysis of the serum, histopathological evaluation of the serum and ^1^H NMR spectroscopic analysis of serum and urine sample. This study was designed based on a previous study and the dose chosen was referred to OECD for testing of chemicals [18,19]. Furthermore, a systematic review on the safety and tolerability of *A. muricata* leaf extract carried out concluded the toxicity effect of *A. muricata* leaf established by 12 previously reported toxicity studies [20]. It is summarized that doses of 155 mg/kg for an aqueous leaf extract and 1092 mg/kg for an ethanolic leaf extract of *A. muricata* were the intraperitoneal LD_50_ values, respectively, conducted in mice models. Meanwhile, the aqueous and methanolic solvents used in rat models showed that the leaf extract was safe at a dose > 5000 mg/kg [16,21,22,23]. Hence, it can be deduced that ethanolic leaf extract was safe within these ranges as methanol is more toxic than ethanol [24,25]. Several studies on *A. muricata* leaf extract toxicity have been performed; however, there is still no study that has been carried out using metabolomics perspectives. NMR-based metabolomics study is a powerful approach to understand further regarding the toxicity of *A. muricata* leaves as this technique had been proven to be one of the most effective methods for examining how organisms react to xenobiotics [18]. In this study, ^1^H NMR metabolomics approach was applied to determine possible endogenous metabolites changes before and after oral administration of *A. muricata* leaf extract. A dose of 2000 mg/kg was chosen since this is the highest dose used to study the effectiveness of *A. muricata* leaf extract in managing various diseases and pharmacological effects.

The results of this research’s phytochemical screening showed the presence of flavonoids, alkaloids, tannins, steroids, triterpenes, and carbohydrates, meanwhile, saponin was absent in this analysis in which the outcome agreed with previous research findings [16,26]. Despite having a therapeutic effect, alkaloids, which are most commonly present in leaves, also appear to be toxic, especially to dopaminergic and other neurons [4,27,28,29,30]. A review of alkaloids and their toxicities detailed the various forms of toxicity brought on by various alkaloids [31]. No significant behavioral changes in the treated group compared to the untreated group after feeding with a single dose of ethanol extract of *A. muricata* leaf until the end of the study.

Moreover, all the rats survived throughout the study. This result was corroborated with the previous studies [32,33] in which no severe signs of toxicity and mortality were identified due to the treatment of *A. muricata* leaf ethanol extract. Furthermore, the weight recorded every two days manifested no significant difference, but the weight of the rats began increasing towards the end of study. In general, this proclaimed that the appetite of the animals was not affected by the extract consumption [18].

A liver function test was carried out to identify the extent of the organ damage caused by the extract. This is because the liver is responsible for the absorption, metabolism, conjugation, and excretion of numerous endogenous and exogenous substances from the body. Hence, any problem and toxicity of the extract will cause high levels of AST, ALP and ALT enzymes. These enzymes will only be released when there is an injury to the liver thus, these enzymes are beneficial for hepatocellular damage markers [34]. The normal levels of ALP, ALT and AST shown in the treated group suggests that no injury occurred. Moreover, kidney function test is conducted to evaluate the overall function of the kidney via the estimation of glomerular filtration rate (GFR). A nitrogen-containing substance called urea, sometimes known as blood urea nitrogen (BUN), is produced in the liver as a byproduct of protein metabolism in the urea cycle. The kidneys remove around 85% of the urea meanwhile the remainder is expelled through the gastrointestinal tract. When renal clearance declines, serum urea levels will rise. Other situations include upper GI hemorrhage, dehydration, catabolic states, and high-protein diets that are unrelated to renal illnesses can also cause a rise in the urea [35]. Urea and creatinine are waste products excreted through the kidney in which high level of these substances in blood indicates that abnormalities are happening in the kidney that prevent it from carrying out its responsibility [36]. The result shown in Table 2 indicated that *A. muricata* leaf ethanol extract at 2000 mg/kg did not affect the levels of these two markers. This result was comparable with a previous study on the ethyl acetate extract from *A. muricata* leaf that showed an LD_50_ of more than 2000 mg/kg in rats [37].

Histopathological examination of *A. muricata* leaf ethanol extract in treated samples of kidney and liver stained with H&E staining showed mild abnormal histological characteristics. The abnormal characteristics found in the liver include mesangial cells hypercellularity, red blood cell congestion and vacuolation between the renal tubules. Hypercellularity of mesangial cells commonly happen due to immune complex disease (ICD) that is usually involved in drug-induced injury. The glomerulus functions as a selective barrier to filter the molecules according to their size, shape, and charge. The filtered plasma may cause injury to the glomerulus that then activates the immune response in the kidney [38,39]. In addition, mild vacuolation in between the renal tubules could be observed in both untreated and treated groups thus, it was not valid to conclude this abnormality occurred due to the administration of *A. muricata* leaf ethanol extract at 2000 mg/kg. Mild vacuolation in between renal tubules indicates the presence of mild hydropic tubular degenerations. Since it appeared in untreated and treated groups, the changes may happen due to the solvent that had been used in this study, Tween 20. Previous study concluded that this solution does have a low toxicity effect [40]. However, the histopathological changes were very mild and insufficiently significant to conclude that Tween 20 had caused significant changes on the renal tubule. Moreover, the concentration of the solvent was based on previous study.

The histology image observed in the liver indicated several mild conditions of liver toxicity aroused since there were mild hemorrhage, mild infiltration of inflammatory cells and dilated central vein with fibrosis. The same characteristics had been found in previous toxicology related studies [41,42]. Mild inflammatory cells infiltration observed may be associated with Kupffer cells. Kupffer cells are macrophages that reside in the liver and act as a first line of defense against particulates and immunoreactive materials. Drug-induced liver injury also can cause the reaction of these cells in which the administration of the extract may trigger Kupffer to respond. However, the presence of Kupffer cells were mild and liver function test also showed no occurrence of liver injury. Hence, it can be concluded that the of the extract did not cause a significant histological change. Numerous medications and chemicals are either produced endogenously by the human body or are introduced from outside compounds that are metabolized and excreted by the liver. The liver is the first place where all of these compounds are catabolized hence, liver injury can occur if toxic substances are being produced [43]. In this study, the biomarker enzymes did not rise during liver and kidney function tests, indicating that the injury was not enough to cause release of the enzymes. The enzymes AST, ALP, and ALT will be released as a result of liver damage [44]. Thus, it can be concluded that the histological changes were very mild and did not cause any significant difference when compared to the untreated group and with other tests. This result was similar to a previous study that showed no histological changes with single dose administration of *A. muricata* leaf ethanol extract at dose 2000 mg/kg [45].

Another objective of this study is to investigate the metabolic profiles after 14 days of administration of ethanolic extract of *A. muricata* leaf. It is also a rapid analytical tool that can be utilized to examine the biochemical variation in biofluids and provide crucial information on herbal toxicity [46]. The results showed no significant metabolites appeared in serum and 15 compounds were identified in the treated group, glucose, trimethylamine *N*−oxide (TMAO), arabinose, ribose, *O*−acetylcarnitine, 2−hydroxyvalerate, isoleucine, *N*,*N*−dimethylglycine, 3,4−dihydroxybenzene, homovanillate, arginine, 5−methoxysalicylate, glycine, 3−methylxanthine and anserine. This means that no significant toxic metabolites had been produced and circulated in the rat’s body. Further analysis on the collected urine was carried out and it was discovered that the urine metabolome profiles of the treated and untreated groups were identical after 14 days of observation. There were also no significant elevated or declined metabolites, indicating that the treated group shared a high similarity of metabolome profiles with the untreated group. The absence of excessive elimination of urea and creatinine from the urine was consistent with the findings of the biochemical tests for renal function. Hence, it can be concluded that *A. muricata* leaf ethanol extract did not cause an acute drastic effect. In the present study, there was no significant changes observed in serum enzymes (ALT, AST, ALP, urea, and creatinine) and ^1^H NMR analysis of serum and urine which further validate the condition of mild changes observed in the kidney and liver histological slides. The metabolome profile of serum and urine supported the result of liver and renal function tests therefore, indicated that the histology observation of the kidney and liver tissues were normal since the changes were not significant. Serum caspase activity is more accurate than alanine aminotransferase (ALT) at identifying early liver damage [28,47]. In addition, a previous study on drug-induced liver injury stated that aminotransferases and alkaline phosphatase, which are often employed as serum indicators of liver injury, are unable to accurately forecast the clinical outcomes of drug-induced liver injury [48]. Further subacute research on histological changes is advised to assess the long-term duration administration of the *A. muricata* leaf ethanol extract on these organs because the histological changes in this study were so minor. The exposure and research periods were relatively brief because the extract was only given in a single dose and the trial was only conducted for 14 days. Furthermore, research on the histological investigation of the liver and kidney in acute, sub-acute, or sub-chronic studies is still limited since most of the studies only focus on the physical observation and mortality of the rats. A sub-chronic study on methanolic extract of *A. muricata* leaf showed a mild noxious effect on the histopathology of the kidney and liver [49]. A study conducted reported that all treatment groups died 24 h after the administration of ethanolic *A. muricata* leaf extract in mice with dose in between 1000–2000 mg/kg [27]. Hence, it is important to determine thoroughly the LD_50_ and toxicity effect of *A. muricata* as this plant is efficient in managing and treating various health conditions but only at an appropriate dosage since this plant does contain toxic compounds in the form of alkaloids [7,50]. Another hypothesis was that the mild histological changes that were observed in the treated group may exist from before the study was conducted as some of the changes also could be found within the untreated group. However, it still can be assumed that *A. muricata* leaf ethanol extract was safe at a dose of 2000 mg/kg. Further research should focus on understanding the possible long-term effects of *A. muricata* leaf ethanol extract. The study’s duration and single-dose administration may not fully capture the potential chronic implications of extract consumption. Extending the exposure and observation period would provide a more comprehensive picture of the extract’s safety profile.

## 5. Conclusions

The findings of this study suggest that a single dose administration of 2000 mg/kg of *A. muricata* ethanol leaf extract does not cause significant acute toxicity signs in rats over a 14-day observation period. Biochemical and advanced ^1^H NMR metabolomics analyses of serum and urine samples did not reveal any significant alterations between untreated and treated groups. Histopathological analyses on kidney and liver organs also did not manifest any significant changes. Given these results, we tentatively propose that *A. muricata* leaf ethanol extract is safe to be used at dose 2000 mg/kg of rat’s body weight. Therefore, the LD_50_ is likely to be more than 2000 mg/kg. Overall, this study suggests that metabolomics is a suitable approach in evaluating the toxicity effect of *A. muricata* leaf extract and it can be applied to other herbal extracts.

## Figures and Tables

**Table 1:** The phytochemical screening tests of *A. muricata*.

Phytochemicals in *A. muricata*	Presences
Flavonoids	+++
Alkaloids	+++
Saponin	−
Tannins	+
Steroids	+
Triterpenes	+
Carbohydrate	+++

Key: (−) Absent (+) present (+++) significantly present.

**Table 2:** Liver function test on the acute oral toxicity of *A. muricata* leaf ethanol extract in female Sprague Dawley rats.

Liver Function Test			
Parameter	Unit	Treatment	
		Untreated Group	Treated Group
ALT	U/L	38.33 ± 8.08 ^a^	40.00 ± 1.00 ^a^
AST	U/L	130.67 ± 25.15 ^a^	134.00 ± 9.54 ^a^
ALP	U/L	99.33 ± 12.58 ^a^	101.00 ± 8.19 ^a^

Values are expressed as mean ± SD by using paired Student’s *t*-test with n = 6 for each group. Same superscript letter (a) means no significant difference from each other (*p* > 0.05). Aspartate aminotransferase (AST); alkaline phosphatase (ALP); and alanine aminotransferase (ALT).

**Table 3:** Renal function test on the acute oral toxicity of *A. muricata* leaf ethanol extract in female Sprague Dawley rats.

Renal Function Test			
Parameter	Unit	Treatment	
		Untreated Group	Treated Group
Creatinine	µmol/L	22.50 ± 2.08 ^a^	25.75 ± 1.50 ^a^
Urea	mmol/L	6.55 ± 0.78 ^a^	6.39 ± 0.90 ^a^

Values are expressed as mean ± SD by using paired *t*-test with n = 6 for each group. Same superscript letter (a) means no significant difference from each other (*p* > 0.05).

## Data Availability

Not applicable.
